# Optical Diagnostic Based on Functionalized Gold Nanoparticles

**DOI:** 10.3390/ijms20184346

**Published:** 2019-09-05

**Authors:** Jiemei Ou, Zidan Zhou, Zhong Chen, Huijun Tan

**Affiliations:** 1 School of Traditional Chinese Medicine Resources, Guangdong Pharmaceutical University, Guangzhou 510006, China; 2 School of Pharmacy, Guangdong Pharmaceutical University, Guangzhou 510006, China; 3 Instrumentation and Service Center for Physical Sciences, School of Science, Westlake University, 18 Shilongshan Road, Xihu District, Hangzhou 310064, China; 4 School of Chemistry, Sun Yat-sen University, Guangzhou 510275, China

**Keywords:** gold nanoparticles, plasmonic, LSPR, SERS, MEF, optical diagnostic

## Abstract

Au nanoparticles (NPs) possess unique physicochemical and optical properties, showing great potential in biomedical applications. Diagnostic spectroscopy utilizing varied Au NPs has become a precision tool of in vitro and in vivo diagnostic for cancer and other specific diseases. In this review, we tried to comprehensively introduce the remarkable optical properties of Au NPs, including localized surfaces plasmon resonance (LSPR), surface-enhanced Raman scattering (SERS), and metal-enhanced fluorescence (MEF). Then, we highlighted the excellent works using Au NPs for optical diagnostic applications. Ultimately, the challenges and future perspective of using Au NPs for optical diagnostic were discussed.

## 1. Introduction

Diagnostics are essential in biomedical science and clinical practice to improve global healthcare. Molecular diagnostic, one of the most important frontiers of modern medicine, relies on the efficient detection of biomarkers extracted from a bio-system to achieve disease diagnosis, evaluation of the disease stage, and monitoring of its progresses and treatment [1,2]. The detection of biomarkers in bio-fluids is promising in point-of-care (POC) applications due to its low invasiveness and high adaptability while the detection of biomarkers in tissues serves as the gold standard for precision diagnosis in pathological examination [3,4,5]. Over the past few decades, much progress has been made in the studies of the detection of biomarkers and their applications in diagnostics [6,7,8,9,10]. However, the sensitive and selective detection of biomarkers in real cases is still challenging owing to the high complexity of biological samples and low abundance of target molecules [2,11,12]. 

Nowadays, immunoassay diagnosis methods based on specific immune responses between antigens and antibodies are relatively mature for clinical diagnosis, which have been applied to hepatitis, pregnancy, venereal disease, tumor detection, etc. Nevertheless, those immunoassay methods, including radiological immunoassay (RIA), immunologic colloidal gold signature (ICS), enzyme-linked immunosorbent assay (ELISA), and chemical luminescence immunoassay (CLIA) have their own advantages and disadvantages. For example, RIA is the most sensitive immunomarking technology with high accuracy and ease of specification and automation. However, the harmfulness of radioisotopes limits its clinical application. ELISA without the need for special instruments and reagents becomes a routine method for clinical diagnosis, but has limitations in precision molecular diagnostics due to its low sensitivity and accuracy. ICS is simple, convenient, and non-polluting but has limited detection range. CLIA is with high accuracy and non-polluting but relatively high cost for detection. Past decades, mass spectrometry (MS) coupled to gas or liquid chromatography (GC or LC) have become the pioneer analytical approaches for the separation, identification, and quantification analysis of biomolecules—i.e., GC-MS and LC-MS. In spite of their high sensitivities and great potentials for online and high-throughput analysis, both methods have their own disadvantages. For GC-MS, identification was based on library spectra and fragmentation patterns, which limits its application in complicated samples. Furthermore, the need for volatility is the major drawback of GC that limits its application for analysis of compounds with molecular weights larger than ca. 600 [13]. Regard to LC-MS, the detection limits often relies on the compound and the instrument used in the experiments [14]. The tandem mass spectrometry coupled to gas chromatography (GC-MS/MS) or liquid chromatography (LC-MS/MS) has largely improved performance in biological analysis by enhancing the analyte identification and reducing the detection and quantification limits. However, these methods are difficult to popularize broadly due to the complicated sample preparation and expensive instruments, which increase the time and cost required for practical applications. Therefore, developments of new strategies for disease diagnosis are needed, as well as improvements to the existing methods. Comparatively, methods based on Au nanoparticles (NPs) can not only improve the sensitivity and accuracy, but also eliminate the dependence on large expensive instruments, which is promising for POC applications.

**Scheme 1:**
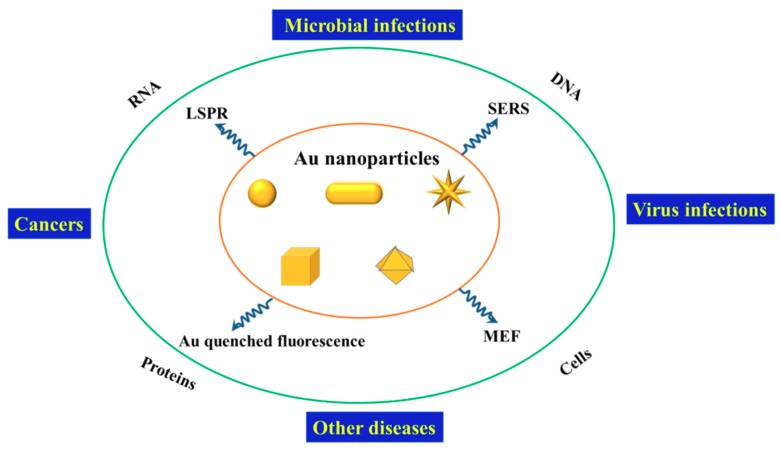
Application of diagnostic spectroscopy utilizing Au nanoparticles.

The advancement of nanotechnology introduced in a broad range of biological applications, in which the engineered noble metal (e.g., gold and silver) nanostructures are very promising in ultrasensitive detection and disease diagnosis due to their unique properties [15,16]. Particularly, Au NPs have become the first choice for researchers owing to their manipulated characteristics (including the sizes, shapes, and opto-electronic properties), favorable biocompatibility, and high stability. To date, Au NPs with various geometric morphologies such as nanospheres [17,18,19], nanorods [17,20], nanostars [21,22,23], nanopyramids [24,25], and nanoflowers [26,27] have been successfully synthesized and explored for biomedical applications. These Au NPs exhibit distinctive and substantial optical properties, including the localized surface plasmon resonance (LSPR), surface-enhanced Raman scattering (SERS), and surface-enhanced fluorescence (SEF), which also called metal-enhanced fluorescence (MEF). The desirable surface plasmonic properties of Au NPs can be achieved by tuning their shapes, sizes, and surface functionalization. Felicitous modification of Au NPs can provide biomarker enrichment and signal enhancement for biomedical detection applications, which has been authenticated by previous researches [18,19,28,29,30]. Nowadays, diagnostic spectroscopy utilizing varied Au NPs has become a precision tool of in vitro and in vivo diagnostic, as described in Scheme 1. Functionalized Au NPs can be used to detect RNA, DNA, proteins, and cells, providing information for the diagnosis of cancers, virus infections, microbial infections, and other specific diseases.

Though the sensitivity and specificity of the biomarker detections have been improving continuously, translation of diagnostic spectroscopy from the laboratory to clinical practice is nontrivial because of some insurmountable obstacles currently, such as the reproducible optical signals, the stability of the Au NPs-based devices in clinical samples and the scarcity of specific biomarkers for distinctive diseases [31,32]. Herein, we tried to comprehensively introduce the remarkable optical properties of Au NPs, including LSPR, SERS, and MEF. Then, we highlighted the excellent works using Au NPs for optical diagnostic applications. Ultimately, the challenges and future perspective of using Au NPs for optical diagnostics were discussed.

## 2. Optical Properties of Au Nanoparticles

**Scheme 2:**
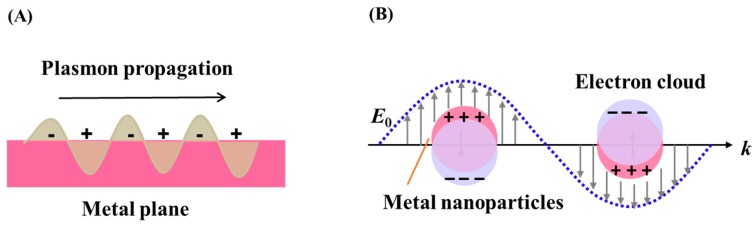
Diagrammatic illustration of the surface plasmon polaritons (**A**) and localized surface plasmon resonance (**B**).

The optical properties of Au NPs rely on the surface plasmon resonance (SPR), which is the coherent oscillation of free electrons at the surface of Au resonate in response to incident light. SPRs can be described as surface plasmon polaritons (SPPs) or localized surface plasmon resonance (LSPR). SPPs originates from propagating waves along a plane metal surface while LSPR occurs when a SP is confined to a volume with dimension smaller than the wavelength of incident light for metal nanoparticles [2,33], as illustrated in Scheme 2. For Au NPs, many applications primarily depend on their LSPR properties, including LSPR sensing, SERS, and MEF.

### 2.1. Localized Surface Plasmon Resonance (LSPR)

Resonant dipolar and multipolar modes in metallic NPs can be excited at specific spectral frequencies by incoming radiation, leading to significant increase absorbed and scattered light and enhancement of electromagnetic fields inside and near the NPs [34]. Metal NPs give rise to both absorption and scattering spectra, therefore their LSPR peaks called extinction spectra. The proportions of the absorption and scattering in the extinction spectra depends on the sizes of NPs that small metal NPs (smaller than 20 nm) essentially show absorption while those with larger sizes show increased ratio of scattering to absorption [35].

**Figure 1:**
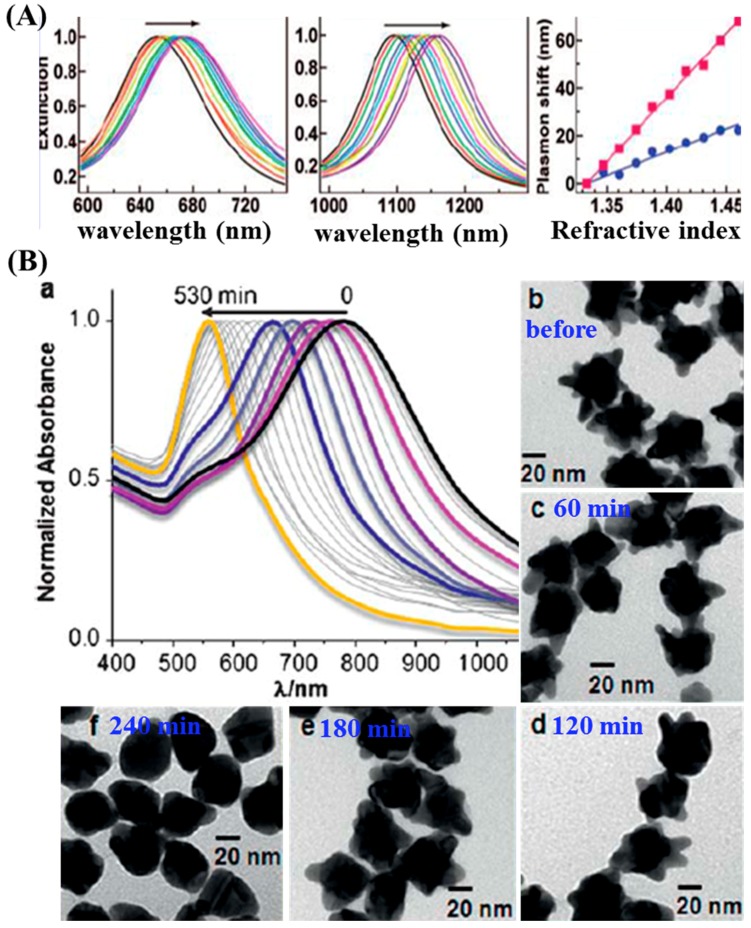
(**A**) Extinction spectra of Au nanorods (aspect ratio = 2.4 ± 0.3), Au nanobipyramids (aspect ratio = 4.7 ± 0.2) in water-glycerol liquid mixtures of varying compositions and dependence of the longitudinal plasmon shift on the refractive index of the liquid mixture for them. Reproduced with permission from reference [40]. (**B**) (a) Spectral time evolution of an AuNS colloid in the presence of cetyltrimethylammonium bromide (CTAB) 0.1 Mm; (b-f) TEM images of Au NSs before mixing and after 60, 120, 180, and 240 min Reproduced with permission from reference [43]. Copyright American Chemical Society, 2008; Copyright Royal Society Chemistry, 2011.

The LSPR peak of Au NPs always ranges in the visible-NIR region, which depends primarily on the size, shape, dielectric environment and composition of the NPs, etc. [36,37]. Au NPs with specific size and desired shape possess tunable properties, resulting in a LSPR peak ranges from 500 nm to 800–1200 nm [38]. Susie et al. tuned the optical properties of Au NPs from 500–1200 nm by changing the NPs from nanosphere of 15–30 nm to nanorods of 2.5–7.5 aspect ratio [39]. Chen and his colleagues explored the refractive index sensitivity of Au NPs with different shapes and sizes, including nanoshpheres, nanocubes, nanobranches, nanorods, and nanobipyramids [40]. Results showed that the response of the LSPR peaks depended on both the shape and size of the Au NPs and the index sensitivities generally increased as Au NPs became elongated and their apexes become sharper, as shown in Figure 1A. 

The classical seed-mediated approach for Au nanostars (NSs) fabrication designed by Liz-Marzan’s group relies on the use of gold seeds, which determining the sizes, shapes, and LSPR properties of the obtained Au NSs. Pazos-Perez et al. systematically investigated influences of the seed concentration, polyvinylpyrrolidone (PVP) concentration, HAuCl_4_ concentration, and reaction time on the plasmon tenability of Au NSs [41]. Results showed that the LSPR peaks could be kinetically controlled and finely tuned in a spectral range from 570 to 870 nm. Additionally, Yuan modified the plasmonic properties of Au NSs by controlling the geometry of Au NSs with varied concentrations of Ag^+^ in the synthesis process [42]. It is found that high concentration of Ag^+^ induced the formations of Au NSs with longer, sharper, and more branches, resulting in red shift of the LSPR band. Au NSs could undergo reshaping into spherical particles by adding a small amount of cetyltrimethylammonium bromide (CTAB), thus allowed fine tuning of LSPR by stabilization of intermediate morphologies during etching process, as illustrated in Figure 1B [43].

Considering the safety, surface functionalization of Au NPs is required before utilizing in biomedical applications. Therefore, Au NPs are usually conjugated with a specific agent such as aptamer, antibody, peptide, and protein for diagnosis studies. Label-free LSPR biosensors mainly rely on the SPR shifts induced by the refractive index changes, which can reach an ultrahigh sensitivity down to the picomolar range [44,45].

### 2.2. Surface-Enhanced Raman Scattering (SERS)

**Figure 2:**
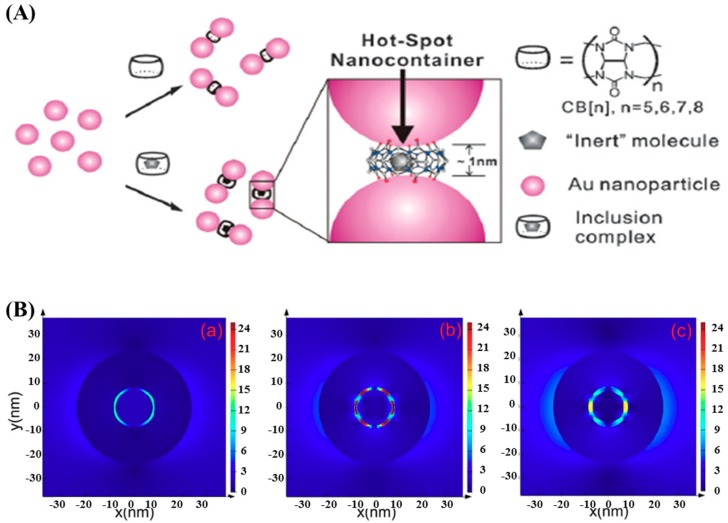
(**A**) Schematic illustration of the fabrication of the SERS hot spot nanocontainer system through bridging Au nanoparticles using Cucurbit (CB) [n] macrocyclic compounds. Reproduced with permission from reference [59]. (**B**) Calculated internal and near-field distributions for nanomatryoshkas (NMs) with 1-nm hollow spherical gap (left), bridged spherical gap (middle), and bridged polygonal gap (right). Reproduced with permission from reference [60]. Copyright Royal Society Chemistry, 2011; Copyright American Chemical Society, 2016.

Raman scattering is the inelastic scattering of a photon by molecules excited to higher vibrational or rotational energy levels. Spontaneous Raman spectroscopy suffers from relatively weak signals because of the small Raman cross-section ~10^−30^ cm^2^/molecule, which limits its applications fundamentally. The first observation of SERS was reported by Fleischmann et al. in 1974 that the Raman scattering signal of a pyridine monolayer layer increased dramatically when it was absorbed on a silver electrode [46]. Subsequently, numerous researchers spared no efforts to investigate the approaches to SERS as well as the enhancement mechanism hidden behind [47,48,49,50]. Enhancement factors ranging from 10^4^ to 10^15^ have been reported in SERS studies [51,52,53,54]. Generally, SERS enhancement can be explained by two mechanisms, the electromagnetic enhancement due to LSPR at roughened metal surface and chemical enhancement due to the charge transfer between metal atoms and molecules [55]. However, numerous experimental results suggest that most contribution is from the electromagnetic enhancement (10^5^~10^8^), and much less (10~10^3^) is from the chemical enhancement [56,57,58]. The electromagnetic enhancement can be further increased by using NPs possessing irregular geometry with spike-like tips, dimers, or assemblies/aggregates with small nanogaps due to plasmon coupling [59,60], as illustrated in Figure 2. Such nanogaps served as hot-spots and dramatically enhanced the signals of the molecules located in them. 

### 2.3. Fluorescence Modified by Au NPs

**Scheme 3:**
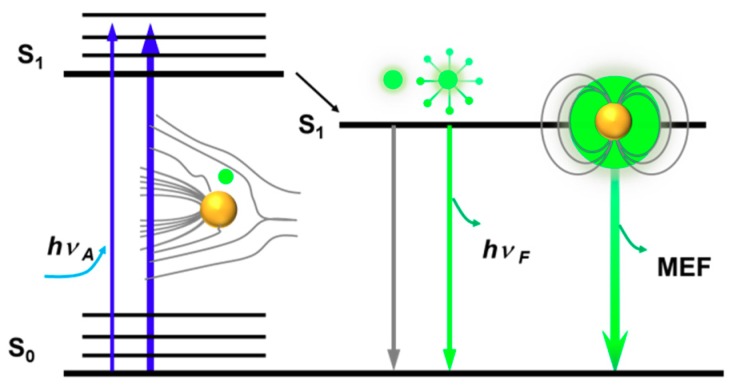
Illustration of the metal-fluorophore interactions by modified Jablonski diagram. The thicker arrows represent the increased excitation (blue) and fluorescence emission (green), respectively.

LSPRs can also couple to the EM fields of emitters in vicinity of the metal NPs, modifying their radiative and nonradiative properties. MEF occurs primarily as a result of the interactions between the excited state of an emitter and the near-field of an excited metal np. Thereby, the distance of the fluorophores from the metallic NPs can affect the SEF process drastically due to the rapid decay of near-fields around the metal NPs [61,62]. A favorable distance within ~30 nm within the metal NPs has been suggested to benefit for near-fields enhancement that both the optical excitation rate and the decay rate for the fluorophore can be increased [63]. Enhancement factors varied from 2 to 1000 can be achieved by MEF. However, when a fluorophore is located too close to the metal NPs (direct contact or with a few nanometers distance), the benefits of LSPR excitation will be eliminated by the domination of nonradiative relaxation rates, resulting to fluorescence-quenching [64,65,66]. The metal–fluorophore interactions can be included in the modified Jablonski diagram (Scheme 3).

## 3. Optical Diagnostic Based on Au Nanoparticles

The sensitivity to the environment of the NPs and interparticles coupling enables the uses of LSPRs in biosensing applications [67,68]. Besides, LSPRs are able to greatly amplify the local electromagnetic (EM) fields, making the Au NPs ideal for use in SERS [69,70,71,72,73,74] and MEF [75,76,77]. The small mode volume of LSPRs also increases the photonic local density of states, which can modify the decay rate and quantum efficiency of emitters in the vicinity of Au NPs [78]. The applications of Au NPs on the molecular diagnostic are based on the spectral modulation.

### 3.1. LSPR Based Diagnostic

The extinction coefficient associated with Au NPs is much higher than conventional dyes, allowing for higher sensitivity and lower limits of detections (LOD) and making them suitable agents for optical sensing applications [79,80,81]. Specially, the shift of LSPR (spectral shift) can be determinate from the color change for the sample solution, which is available for naked-eye readout. For instance, the “dispersion-to-aggregation” state of Au NPs is displayed with a “red-to-blue” color shift (spectral red shift), making it possible for many sensing applications by naked-eye readout. Upon selectively binding to target molecules like DNA strands or proteins, the dielectric environment near the surface of Au NPs is altered. Consequently, the binding process can be monitored via the change of LSPR peaks.

Enzyme-linked immunosorbent assay (ELISA), a routine method for screening of disease, has limitation in precision molecular diagnostic due to its low sensitivity and accuracy. Owing to their high surface area-to-volume ratio, Au NP-enhanced ELISA allows much more attachment of antibody-enzyme complexes and then overcomes the limitation of conventional ELISA mentioned previously. Great progress in developing Au NP-based plasmonic sensors for ELISA have achieved by means of enzyme-mediated growth or aggregation of Au NPs [82,83].

**Figure 3:**
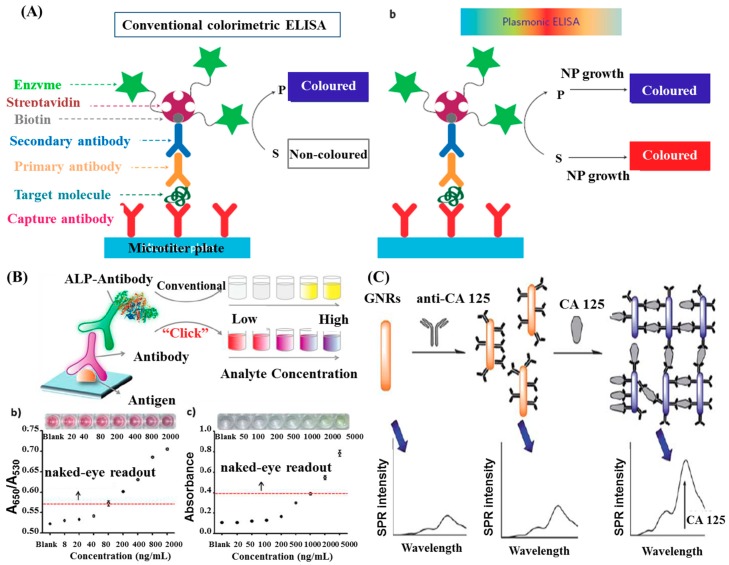
(**A**) Schematic representation of the sandwich ELISA format used here and two possible signal generation mechanisms (S, substrate; P, product; NP, nanoparticle). Reproduced with permission from reference [82]. (**B**) A comparison between CuAAC-mediated plasmonic ELISA and conventional ELISA. Reproduced with permission from reference [84]. (**C**) Schematic representations of CA 125 detection using plasma resonance scattering properties of gold nanorods. Reproduced with permission from reference [6]. Copyright Springer Nature, 2012; Copyright American Chemical Society, 2014; Copyright Royal Society Chemistry, 2013.

A plasmonic enzyme-linked immunosorbent assay (ELISA) for detection of disease biomarkers at ultralow concentrations was introduced by de la Rica et al. [82]. The enzyme label of the ELISA controlled the formation of Au NPs and generated colored solutions of characteristic distinct tonality (Figure 3A). The detections of prostate specific antigen (PSA) and HIV-1 capsid antigen (p24) were performed in whole serum at ultralow concentration down to 1 × 10^−18^ g/mL. This plasmonic ELISA did excellent work in the detection of p24 in the sera of HIV-infected patients. Recently, Ruiz-Sanchez et al. developed a tunable plasmonic gold dendrimer nanochain immunoassay and employed it to the POC diagnostic for p24 in pseudo-serum [6]. The LOD of 5 ng/mL was 4-fold more sensitive than comparable studies with Au NPs. These findings and creative concepts highlight the potential of early diagnosis for HIV infections.

Though Au NP-based plasmonic sensors have extensively exploited for ELISA applications, their direct impose on the current ELISA platform is still the main challenge. To tackle this problem, Xianyu et al. developed a Cu (I)-catalyzed azide/alkyne cycloaddition (CuAAC)-mediated plasmonic nanosensor for immunoassay via enzyme-triggered click chemistry, where the alkaline phosphatase-triggered click chemistry between azide/alkyne functionalized Au NPs acted as the readout [84]. The authors employed this CuAAC-mediated plasmonic nanosensor to test the rabbit antihuman IgG with concentrations ranging from 0.8 ng/mL to 2000 ng/mL and observed a gradual color change from red to blue of the Au NPs solution with increasing target proteins (Figure 3B). Comparatively, this CuAAC-mediated plasmonic nanosensor enabled a naked-eye diagnosis of mycoplasma pneumonia (100%), whereas conventional ELISA is not capable of distinguishing by the naked eye in some cases.

The work by Wang et al. reported a reverse phenomenon that the presence of the targeted telomerase inhibited the aggregation of Au NPs, resulting blue shift and narrowing of the LSPR band [85]. The aforementioned colorimetric assay fabricated with primer-modified Au NPs can measure telomerase activity down to 1 HeLa cell/uL.

Zhang et al. proposed a novel strategy for analysis of cancer antigen 125 (CA 125) using the plasmon resonance scattering properties (the transversal absorption band) of Au NRs [86], as illustrated in Figure 3C. The immunoreaction between the prepared Au NR-anti-CA125 conjugates and CA 125 caused aggregations of Au NRs, allowing determination of the target protein concentration by analyzing the plasmon resonance scattering spectra. At a concentration range of 1.0–80 U/mL, the detection limit of 0.4 U/mL could be achieved.

Apart from cancers and virus, microbial infections can be also lethiferous. Au NP-based ELISA could be used to quantitative assay *Treponema pallidum* (*T. pallidum*) at about 1 pg/mL level, which was 1000-fold improvements over a conventional ELISA [83]. Bacteria-instructed click chemistry between functionalized Au NPs was employed for POC microbial sensing, e.g., *Escherichia coli* [87]. The colorimetric strategy could be easily integrated in a smartphone app as a portable platform. This will be the significant objects in the future.

### 3.2. SERS Based Diagnostic

SERS is a very attractive spectroscopic technique in biomedical applications because of its non-invasive and high sensitivity features. An amplification increase of the Raman signal by a factor greater than 10^6^ has been reported by numerous researches, and the enhancement factor can reach up to 10^14^–10^15^ times in hot-spots [20,35]. These enhancements improve the detection limit from ensembles of molecules to the single molecule level, making SERS an extremely useful tool for biomolecular detection.

**Figure 4:**
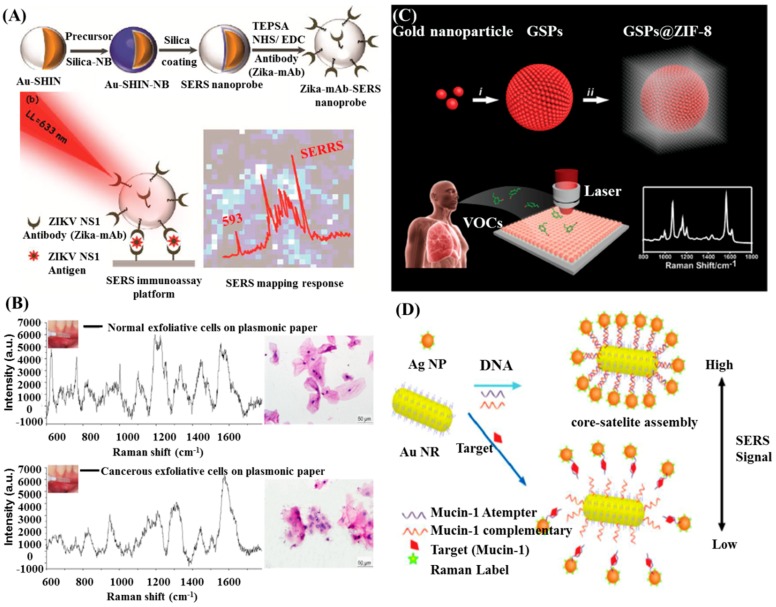
(**A**) Illustration of the SERS immunoassay platform for detecting ZIKV NS 1. Reproduced with permission from reference [91]. (**B**) Cancerous cells screening using the paper-based SERS platform. Reproduced with permission from reference [92]. (**C**) Diagrammatic sketch of synthesizing GSP@ZIF-8 core-shell structure and the volatile organic compounds (VOCs) detection via SERS spectroscopy. Reproduced with permission from reference [93]. (**D**) Scheme of SERS aptasensor for the detection of Mucin-1based on Au NRs-Ag NPs core-satelliteassemblies. Reproduced with permission from reference [94]. Copyright American Chemical Society, 2018; Copyright Elsevier, 2014; Copyright John Wiley & Sons, Inc, 2017; Copyright Royal Society Chemistry, 2015.

Spherical Au NPs are the most commonly used SERS substrates [88,89]. Wu et al. proposed a direct detection of circulating tumor cells (CTCs) in the blood sample with a small LOD of 5 cells/mL by using Au NPs as the SERS substrates [90]. Camacho et al. reported an immunoassay based on shell-isolated nanoparticle-enhanced Raman scattering (SHINERS) for detection of zika virus (ZIKV) with a LOD of ZIKV NS1 down to 10 ng/mL (Figure 4A) [91]. In their work, ultrathin silica shell was acquired to enclose the Raman reporter and avoid uncontrolled aggregation of the nanoparticles, improving the detection sensitivity. 

The enhancement of SERS signals usually relies on the intensity of electromagnetic filed, which can be further enhanced by plasmon coupling. It has been proved that non-spherical, irregular NPs induced much stronger electromagnetic filed than that of spherical, regular NPs [17]. Thereby, non-spherical gold nanostructures—such as nanorods, nanostars, nanowire, and nanoprisms—have been fabricated and explored for SERS applications. Among of these nanostructures, gold nanorods (AuNRs) receive much attention because of their unique optical properties with a longitudinal SPR and a transverse SPR band in extinction spectra. The tunable longitudinal SPR band and strong enhancement at the tips makes Au NRs suitable for SERS application. A paper-based plasmonic platform consisting of Au NRs could be used to sensitive, noninvasive, and rapid cancer screening [92]. Different SERS spectra were obtained from the normal and cancerous cells, which could be analyzed by a diagnostic algorithm to distinguish these cells, as shown in Figure 4B. Raman spectroscopy combined with multivariate statistical methods had been also used to distinguish different cells. However, Raman spectra of a biological sample are very complex that the spectral differences are often minute and difficult to identify. The separation achieved by this method needs many repeated measurements to get enough data and requires a long time to extract useful information, which is not suitable for real clinical diagnosis. 

As mentioned above, assembly of Au NPs can enrich molecules in the nanogaps. Qiao et al. proposed a strategy to selectively and sensitively detect gaseous biomarkers from lung malignancies patients’ exhalation by using a self-assembly of gold superparticle coating with ZIF-8 layer (GSPs@ZIF-8) (Figure 4C) [93]. Gaseous aldehydes, the indicators of lung cancer, were selectively captured by the GSP substrate via the Schiff base reaction with the Raman-active probe molecule p-aminothiophene (4-ATP). Then, analysis of the SERS signal from 4-ATP could provide information correlated to the aldehyde volatile organic compounds (VOCs) concentrations.

Bimetallic nanostructures can further improve the SERS activity of Au NRs. Wu et al. reported the utilization of gold/silver core-shell nanorods (Au@Ag NRs) in immunoassay and founded that Au@Ag NRs exhibited elevated SERS activity than uncoated Au NRs [95]. The authors modified the Au@Ag NRs with antibody and then immobilized them on glass slide to fabricate an immunoassay for human IgG detection. SERS measurements revealed a concentration-dependent manner of human IgG in the range of 700 nM to 70 fM. The detection limit of this kind of immunoassay reached 70 fM, about 104 times lower than uncoated Au NRs based detection. Feng et al. established an ultrasensitive aptamer-based SERS sensor for the detection of Mucin-1, a specific breast cancer marker protein, using Au NRs core-Ag NPs satellite assemblies, as illustrated in Figure 4D [95]. The core-satellite assemblies started to release in the presence of Mucin-1, leading to decreased Ag NPs around the Au NRs core and then SERS intensity. The limit of detection (LOD) for Mucin-1 was 4.3 aM. Practical analysis of Mucin-1 in human blood serum revealed the reliability of this sensor, indicating that this method is feasible and promising for the early monitoring of breast cancer.

Besides Au nanospheres and Au NRs, multibranched Au NSs have also attracted much attention because of their optical tunability by engineering subtle changes in geometry. The sharp branches on Au NSs can create a ‘lighting rod’ effect, enhancing the local EM field dramatically [96]. Meanwhile, these multibranched nanostructures have increased surface-to-volume ratios, allowing more Raman reporters to attach onto their surface. Moreover, the hybridization of the individual tips of the Au NSs could serve as hot-spots and further enhance SERS signals [31]. 

**Figure 5:**
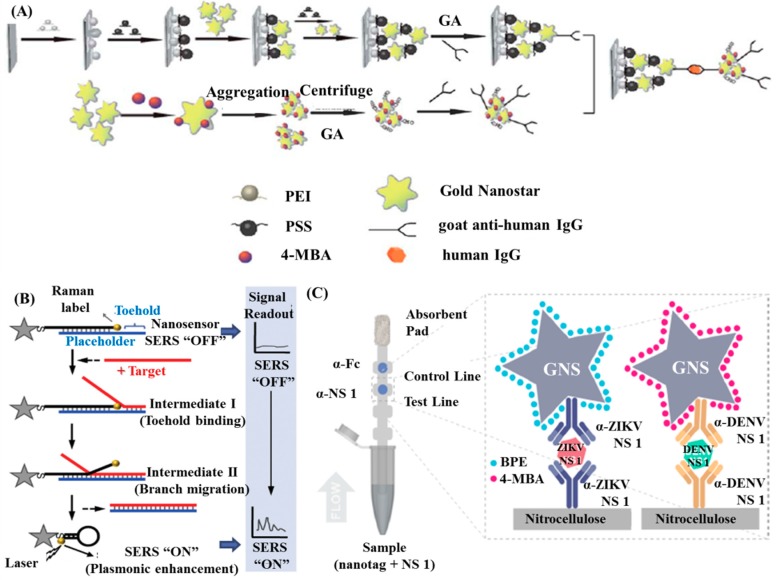
SERS-based immunoassay using Au NSs: (**A**) Schematic diagram of the synthesis route of immune nanostar aggregates and SERS-active substrates. Reproduced with permission from reference [97]. (**B**) Detection scheme of the ‘turn on’ SERS nanobiosensor. Reproduced with permission from reference [98]. (**C**) Sandwich immunoassay for ZIKV and DENV NS 1. Reproduced with permission from reference [99]. Copyright Royal Society Chemistry, 2013; Copyright Elsevier, 2015; Copyright American Chemical Society, 2017.

Pei et al. established a SERS-based sandwich immunoassay by combining use self-assembled Au NSs and aggregates of Au NSs, as shown in Figure 5A [97]. The detection of human IgG antibodies was performed at the concentration region 10^−4^ to 10^−14^ g/mL, exhibiting a linear correlation between the concentration of antigens and the prominent SERS signals. The small LOD was as low as 10 fg/mL, prompting wide-range applications in bio-detection with such an immunoassay. Wang et al. described a new conceptual “turn on” plasmonic-based nanobiosensor for homogeneous nucleic acid detection [98]. As shown in Figure 5B, the nanobiosensor was composed of a “stem-loop” DNA probe with a Raman label at one end and a single-stranded DNA serving as a “placeholder” strand that partially hybridized to the stem-loop probe. In the absence of target, the ‘placeholder’ strand separated the label away from the Au NSs surface, providing low SERS intensity (off status). In contrast, the “placeholder” strand got rid of the Au NS surface following a non-enzymatic strand displacement process. This displacement process allowed the stem-loop structure to ‘close’ and moved the Raman label onto the Au NS surface, yielding a strong SERS signal (“on” status). This kind of nanosensor with a LOD of ~ 0.1 nM is efficient for nucleic acid diagnostic. 

In recent research by Sánchez-Purrà et al., a multiplexed SERS-based immunoassay were designed by combining lateral flow assay (LFA) with SERS and utilized for detection of Zika virus (ZIKV) and dengue virus (DENV) [99]. In that immunoassay, Au NSs SERS-encoded with 1,2-bis(4-pyridyl)ethylene (BPE) and 4-mercaptobenzoic acid (MBA) were conjugated to specific antibodies for both diseases, owing the ability to distinguish ZIKV and DENV nonstructural protein 1 (NS 1) biomarkers, as illustrated in Figure 5C. 15-fold and 7.2-fold lower LOD relative to colorimetric for ZIKV NS 1 and DENV NS 1 were determined from the immunoassay tests respectively.

Having advantages of fingerprint vibrational signals with ultra-narrow line widths, SERS-activated nanoparticles have found optical coding and multiplexing immunoassay applications. Previously, Cui’s group concluded the impressive progress in SERS immunoassay, including the design and fabrication of SERS nanoprobe, SERS-encoding techniques and applications for specific multiplex detections in an immunoassay system [31]. Thereby, the multiplexing immunoassay based on coding SERS will not be discussed here.

### 3.3. Fluorescence Based Diagnostic

Diagnostic based on the fluorescence-quenched by Au NPs are always designed as “turn-on” detection exploiting the universal quenching effect ability of Au NPs. With regard to this type of applications, probes based on oligonucleotide-functionalized Au NPs have attracted much attention. 

**Figure 6:**
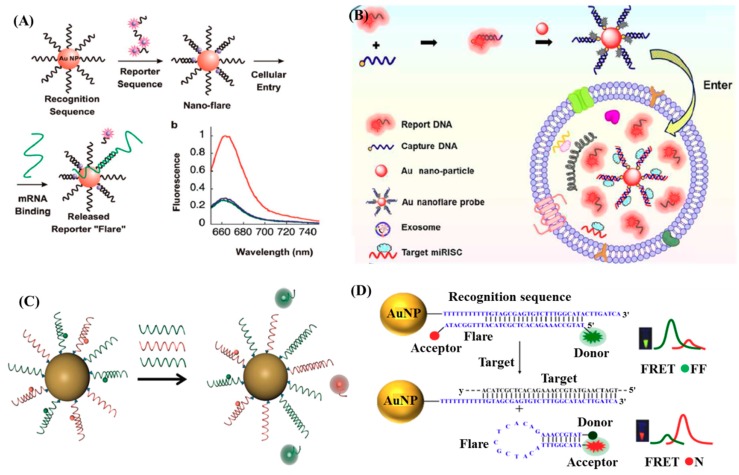
(**A**) Nanoflares probes for mRNA detection. Reproduced with permission from reference [105]. (**B**) The structure of Au nanoflare probe and its mechanism of detecting miR-1246 in exosomes. Reproduced with permission from reference [110]. (**C**) Schematic representation of target detection by multiplexed nanoflares. The multiplexed nanoflares bind different target nucleic acids (shown in red and green), displacing the corresponding flare. Reproduced with permission from reference [102]. (**D**) Design and working principle of the specific sequence responsive FRET nanoflares. Reproduced with permission from reference [107]. Copyright American Chemical Society, 2007; Copyright American Chemical Society, 2018; Copyright American Chemical Society, 2012; Copyright American Chemical Society, 2015.

As proved by many researchers, endogenous mRNA can be a specific indicator of disease that its expression level can provide plentiful information about the disease progression and prognosis [100,101]. Therefore, the identification and characterization of disease-related mRNA in cells is of great significance for early diagnosis and treatment of numerous diseases (e.g., cancer). However, the conventional approaches—such as reverse transcription polymerase chain reaction (RT-PCR), Northern-blots, and microarray analysis—are limited by the requirement for groups of cells and incapable of analysis of single cells in real time [102,103]. Oligonucleotide-functionalized Au NPs have high intracellular delivery efficiency by endocytosis, high stability, and high signal-to- background ratios for mRNA detection in living cells [104,105]. Nanoflare, a new class of probe, has exhibited great promise in intracellular mRNA detection as well as labeling and isolating specific cells since its first appearance in 2007 [102,103,105,106,107,108]. A typical nanoflare is a spherical nucleic acid Au NP conjugates consisting of densely packed recognition oligonucleotides with complementary sequences to the target mRNA, many of which are hybridized to the so-called flare oligonucleotides with fluorophore labeling. The nanoflare functionalized with oligonucleotides is a polyanionic structure that can effectively enter the cell via endocytosis, and therefore be capable of detecting intracellular mRNA levels, as illustrated in Figure 6A [105]. The rapid cellular uptake kinetics and intracellular transport of nanoflares stem from the arrangement of oligonucleotides into 3D architecture, which supports their targeting of scavenger receptors and endocytosis via a lipid-raft-dependent, caveolae-mediated pathway [109]. To date, nanoflare has been successfully applied to not only the detection of intracellular mRNA, but also the detection, isolation, and culture of live cells. Halo et al. reported the first genetic-based approach for fluorescently detecting, isolating, and analysis of live circulating tumor cells (CTCs) from blood by coupling the nanoflares with flow cytometry [106]. Though the LOD of 100 cells/mL is much higher than the real concentration of CTCs in real cases, this technique provides a new opportunity for cancer diagnosis, prognosis, and therapy. 

Though the amazing capability of the nanoflare has been proved through the successfuldetections of mRNA and live cells, the single-signal-based sensing may be compromised by the distribution of probes and the drifts of the instruments, hindering the quantification of the relative concentrations of different targets and generating false positive signals due to the thermodynamic fluctuations. To overcome these limitations, Prigodich et al. developed a kind of multiplexed nanoflares capable of simultaneously detecting two mRNA targets, as shown in Figure 6C [102]. The polyvalency of the oligonucleotides within the nanoflare allows multiple capture sequences included on a single nanoparticle, which enable multiplexed detection. Besides, Yang et al. developed a new and upgraded nanoflare using two-fluorophore-labeled ‘flares’, which could be used for ratiometric fluorescent measurement based on fluorescence resonance energy transfer (FRET) [107]. As illustrated in Figure 6D, the Au NP functionalized with recognition sequences hybridized to flares, which were designed as hairpin structures and fluorescently labeled donors (FAM) and acceptors (TAMRA) at their 5’ and 3’ termini, respectively. In absence of targets, the flares were bound with the recognition sequences, separating the donor and acceptor and leading to low FRET efficiency with the fluorescence of donors as the only output. In contrast, the presence of targets induced the displacement of the flares away from the recognition sequences and the formation of hairpin structures gradually, promoting the donor and acceptor in close proximity to each other and then resulting in high FERT efficiency. In this situation, the fluorescence of the acceptor can be output.

Live detection of mRNAs in cells using nanoflares attracts much interest from scientists. The commercial version of the nanoflares (named as smartflares) have been commercially available. However, several publications shown that nanoflares cannot possibly detect the intracellular mRNA level because of the lack of correlation between mRNA levels and nanoflare fluorescence [111,112,113]. On one hand, cells with different types or under different conditions might endocytose different amounts of nanoflares, yielding varied fluorescence signals. On the other hand, nanoflares could be degraded in the presence of DNAse I [114]. Furthermore, the endosomal escape needs to be taken into account that only the escaped particles can detect cytosolic mRNAs. These disputes inform our understanding of the potential and limitations of nanoflares.

Compared with spherical Au NPs, Au NRs exhibit favorable absorption in the near-infrared (NIR) region, leading to enhancement of the quenching effects that available for NIR range bioassays. A luminescence energy transfer (LET) system constructed by using NIR-to-NIR upconversion lanthanide nanophosphors (UCNPs) as donor and Au NRs as acceptor has been applied to the detection of thrombin in aqueous buffer and human blood samples [115]. This LET system exhibited LODs for thrombin as low as 0.118 nM and 0.129 nM in buffer solution and human serum, respectively.

Au nanostructures can significantly enhance the fluorescence from nearby fluorophores, making nanoprobes consisting of Au NPs an excellent contrast agent for bioimaging. Zhao et al. demonstrated the enhanced two-photon excitation fluorescence of photosensitizers using a core-shell nanostructure with Au NRs [116]. The separation distance between the Au NRs core and the porphyrin molecule (T790) was adjusted by varied thickness of the silica shell. In the optimum conditions, enhancement factors of 2.1 and 11.8 were obtained for one- and two-photon excitation fluorescence, respectively. Subsequently, these NPs were used as effective agent for two-photon cell imaging on cancer cells. In fact, the NIR fluorescence enhanced molecular imaging of live cells was earlier reported by Hong et al. that molecular imaging of cells in the 0.8 to 1.4 μm spectral window was perform based on labelling with both carbon nanotubes and organic fluorophores [117].

Apart from enhanced substrates, Au NPs can act as imaging agents due to their inherent luminescence properties. Gold nanoclusters (Au NCs) have been emerged as a new class fluorescent materials for various bioanalysis. The Au NCs often consist of several to tens of atoms with diameter below 2 nm. With this small size, Au NCs exhibit discrete, size-tunable electronic transitions, and molecular-like properities instead of SPR properties because of the quantum confinement effect [118]. Thus, fluorescent Au NCs can be utilized for bio-labeling, bio-imaging, and bio-detection. Wang et al. developed a transferrin functionalized gold nanoclusters/graphene oxide (Tf-AuNCs/GO) nanocomposite as a turn on NIR fluorescent bioimaging of cancer cells and small animals [119]. The prepared Tf-AuNCs exhibited strong NIR fluorescence, which could be quenched by the GO in the nanocomposite and restored by addition of the transferrin receptor (TfR) effectively.

### 3.4. Dual-Mode/Multiplex Diagnotics

**Figure 7:**
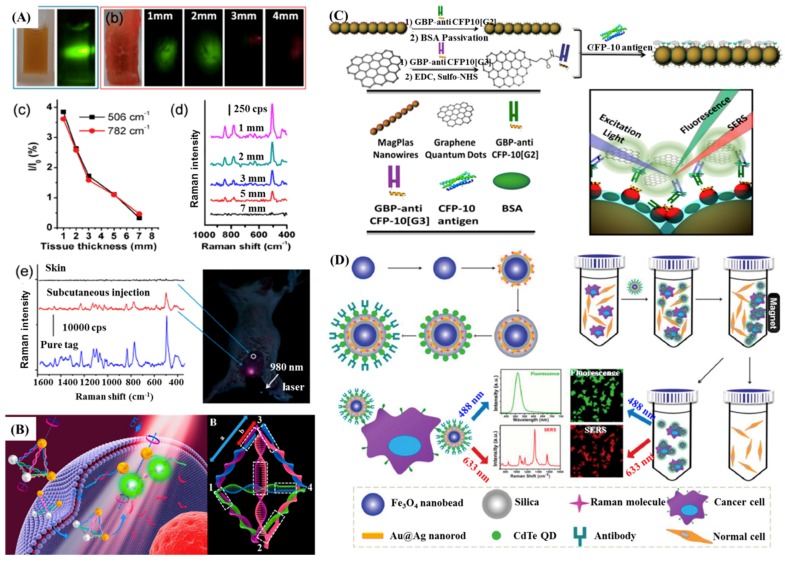
(**A**) Bioimaging applications of UCF-SERS tags. Reproduced with permission from reference [121]. (**B**) Working principle of Au-UCNP pyramids for miRNA detection and the nucleic acid skeleton of pyramid used for miRNA detection. Note: 3, 4 are linked with Au NPs; 1, 2 are linked with UCNPs. Part a: Recognition sequence of miRNA; and part b: non-complemented part (circled by white dashed line). Reproduced with permission from reference [24]. (**C**) Schematic representation of the synthesis of CFP-10 antibody (G2 or G3)-functionalized Fe_3_O_4_@PEI@Au nanowires (MagPlas NW) and GQD nanocomposites and (lower right) the detection mechanism of the SERS-induced immunoassay to monitor the target CFP-10 antigen. Reproduced with permission from reference [122]. (**D**) Schematic illustration of the fabrication of the nanoprobe, the process of cancer cells with specific receptor on their surfaces captured by the nanoprobes and the cell separation procedures. Reproduced with permission from reference [123]. Copyright American Chemical Society, 2014; Copyright American Chemical Society, 2016; Copyright American Chemical Society, 2015; Copyright John Wiley & Sons, Inc., 2014.

Although Au NPs-based SERS and MEF assays have shown promising application in various fields, they are still limited by their instinct shortcomings, the slow imaging speed for Raman scattering and the obstacle of multiplex detections for fluorescence. However, combining SERS or MEF with other techniques will help in overcoming those problems in many biomedical applications. For instance, SERS-fluorescence dual probes can be used to ultrasensitive multiplex detections and biomedical imaging simultaneously. Lee et al. fabricated SERS-fluorescence dual modal nanoprobes (DMNPs) and investigated their application in imaging for duplex co-expressed markers on cancer cells [120]. It was found that SERS imaging with DMNPs could determine the localized spatial distributions of cancer markers in living cells, having great promise to application of early cancer diagnosis. Upconversion fluorescence (UCF)-SERS dual mode tags were also designed for cellular and in vivo imaging, in which silica coated NaYF4: Yb, Er upconversion nanoparticles (UCNPs) served as the fluorescent core [121]. As illustrated in Figure 7A, signals of the UCF and SERS could be detected from 3 and 7 mm deep pork tissues in ex vivo experiments. Then, results of in vivo imaging on live mice further verified the capabilities in bioimaging of UCF-SERS tags.

Li et al. fabricated a dual-mode nanoprobe using DNA driven Au-UCNP pyramids to detect intracellular microRNA (miRNA) in real time, as illustrated in Figure 7B [24]. The Au-UCNP pyramids had strong plasmonic circular dichroism (CD) and fluorescence signals, allowing monitoring by both of them. The chiroplasmonic nanopyramids showed a remarkable linear range from 0.073 to 43.65 fmol/10 μgRNA and a LOD of 0.03 fmol/10 μgRNA for the CD measurements, and a range from 0.16 to 43.65 fmol/10 μgRNA with a LOD of 0.12 fmol/10 μgRNA for the fluorescent measurements.

Zou et al. designed a novel dual-mode SERS-fluorescence immunoassay using graphene quantum dot labeling on one-dimensional aligned magnetoplasmonic nanoparticles to monitor a tuberculosis (TB) antigen, CFP-10, as illustrated in Figure 7C [122]. The mentioned dual-mode nanoprobes with a low LOD of 0.0511 pg/mL are expected to further used to high throughput screening of targets due to their easy operation and superior multiplexing ability of the immunoassay protocol. The introduction of magnetic core to SERS-fluorescence dual mode nanoprobes even enabled them to recognize and capture various cancer cells specifically, as described in Figure 7D [123].

## 4. Conclusions and Future Perspective 

Thanks to their unique physical and optical properties, Au NPs engineered with desired properties have been extend to the trace analysis of targets, exhibiting superior performance in many biomedical application, including molecular recognition, sensing, and imaging. Functionalized Au NPs can be bound to the specific targets (RNA, DNA, and proteins), thus being capable of the detections of biomarkers that related to specific diseases, e.g., cancers, lethiferous infections, and chronic diseases. Moreover, functionalized Au NPs exhibit excellent biocompatibility and low toxicity having great potential application in real clinical diagnosis. The plasmonic based optical properties of Au NPs—included LSPR, SERS, MEF, etc.—have been applied to the development of various kinds of biosensors and detection platforms for diseases diagnosis. LSPR based diagnostic mainly relies on the spectral shift (red-shift usually) of the plasmonic peaks, which also reflects as color changes of the assayed solutions. Therefore, this kind of color change is easily readout with naked-eye in some ranges. Expanding the spectral shift along with the changes of target concentrations will enhance the visualization from the color changes. SERS based diagnostic is ultrasensitive that enabling single molecule detection. The sharp and narrow characteristic Raman peaks give great potential for multiplex detection via an optical coding strategy. As for fluorescence modified by Au NPs, both fluorescence-quenching and fluorescence-enhancement have been employed to biological applications. The most obvious advantages of the fluorescence-based diagnostic is its prominent imaging ability that can be used in vivo. Combining these techniques with each other or other technique (e.g., magnetic beads) can achieve multi-mode diagnostic and then further improve the performance of Au NPs in disease diagnosis. By using these methods, the detection of disease related biomarkers can be down to ultralow concentrations with LODs at fg/mL ~ pg/mL levels. 

So far, translation of those approaches from laboratory to clinical practice is nontrivial because of some insurmountable obstacles currently. Firstly, non-specific binding to non-targeted molecule induces interference or false information to the detection, being the biggest problem to be resolved. In physiological conditions, the specific target molecules are nearly equivalent to other biomolecules, leading to inevitable non-specific absorption of non-targeted molecules. The competition between the target molecules and non-target molecules limits the quantitative analysis, thus affecting the accuracy of disease diagnosis. Secondly, optical signals from the Au NPs-based immunoassay are not reproducible because of the heterogeneity of metal substrates in many cases. Most Au-based nanoprobes are fabricated via chemical methods, whose sizes and shapes could be varied due to the differences in the synthesis process, such as the quality of original materials, temperature, pH, and mixing speed. Developments of the processing technique like electron beam lithography allows for the fabrication of reproducible metal substrate with uniformed size and shape—e.g., nanoarrays [124]—improving the mentioned problem to an extent. However, the expensive equipment and cost cannot be affordable for republic institutions, hindering its use in practical application at present. With regard to DNA-programmed assemblies, one drawback also exists that the location of the DNA molecule on a NP’s surface cannot be easily controlled. Thirdly, the complex components in the clinical samples (blood, sweat, urine, etc.) could cause the aggregation of the nanoprobes, leading to significant variations in optical signals. The potential toxicity and unknown metabolism pathway prevent people from venture in vivo applications. 

Translation of Au NP-based optical diagnostic into clinics should begin with the modification of existing methods like ELISA. Au NPs emerged into paper-based immunoassay platform opens a predictable way for POC applications. Last decade, Au NPs have been introduced to microflow-controlled chips, which can improve the ability to identification, isolation, and detection of targets. Follow with the intelligent process, portable devices can be developed by using smartphone as data-output equipment. Thereby, the goal of the development of Au NP-based optical diagnostic will primarily focus on POC applications in the future.

Undoubtedly, great efforts are still needed to promote Au NP-based optical approaches from proof-of-concept research to commercially available analytical tools. Nevertheless, the drawbacks cannot obstruct the functionalized Au NP-based optical spectroscopy become one of the most potential tool for disease diagnosis. Those problems would be solved with the advanced development of the nanotechnology, micro-nano processing technology and biomedicine. Au NP-based optical diagnostic has great future perspective in early diagnosis of cancers, infections, and other diseases.
